# Measures of financial toxicity in cancer survivors: a systematic review

**DOI:** 10.1007/s00520-024-08601-4

**Published:** 2024-06-04

**Authors:** L. B. Thomy, M. Crichton, L. Jones, P. M. Yates, N. H. Hart, L. G. Collins, R. J. Chan

**Affiliations:** 1 Division of Cancer Services, Princess Alexandra Hospital, Metro South Health, Ipswich Road, Woolloongabba Q4102, Brisbane, QLD Australia; 2 Caring Futures Institute, College of Nursing and Health Sciences, Flinders University, Adelaide, SA Australia; 3 QIMR Berghofer Medical Research Institute, Brisbane, QLD Australia; 4 Cancer and Palliative Care Outcomes Centre, School of Nursing, Faculty of Health, Queensland University of Technology (QUT), Brisbane, QLD Australia; 5 Centre for Healthcare Transformation, Faculty of Health, Queensland University of Technology (QUT), Brisbane, QLD Australia; 6 Human Performance Research Centre, INSIGHT Research Institute, Faculty of Health, University of Technology Sydney (UTS), Sydney, NSW Australia; 7 Exercise Medicine Research Institute, School of Medical and Health Sciences, Perth, WA Australia; 8 Institute for Health Research, University of Notre Dame Australia, Perth, WA Australia; 9 Australia School of Public Health, The University of Queensland, Brisbane, QLD Australia

**Keywords:** Financial toxicity, Distress, Hardship, Cancer, Psychometrics

## Abstract

**Purpose:**

Comprehensive cancer-related financial toxicity (FT) measures as a multidimensional construct are lacking. The aims of this systematic review were to (1) identify full measures designed explicitly for assessing FT and evaluate their psychometric properties (content validity, structural validity, reliability, and other measurement properties) using Consensus-Based Standards for the Selection of Health Measurement Instruments (COSMIN), and (2) provide an analysis of the domains of FT covered in these measures.

**Methods:**

MEDLINE, CINAHL, Web of Science, and Cochrane CENTRAL were searched for quantitative studies published from January 2000 to July 2023 that reported psychometric properties of FT measures in cancer survivors. The psychometric properties of FT measures and study risk of bias were analysed using COSMIN. Each FT measure was compared against the six domains of FT recommended by Witte and colleagues. Results were synthesized narratively. The detailed search strategies are available in Table S1.

**Results:**

Six FT tools including the COST-FACIT, PROFFIT, FIT, SFDQ, HARDS, and ENRICh-Spanish were identified. The COST-FACIT measure had good measurement properties. No measure reached an excellent level for overall quality but was mostly rated as sufficient. The SFDQ, HARDS, and ENRICh-Spanish were the most comprehensive in the inclusion of the six domains of FT.

**Conclusion:**

This review emphasizes the need for validated multidimensional FT measures that can be applied across various cancer types, healthcare settings, and cultural backgrounds. Furthermore, a need to develop practical screening tools with high predictive ability for FT is highly important, considering the significant consequences of FT. Addressing these gaps in future research will further enhance the understanding of FT.

**Supplementary Information:**

The online version contains supplementary material available at 10.1007/s00520-024-08601-4.

## Introduction

Financial toxicity (FT) has emerged over the last two decades as the negative impact of direct and indirect costs linked to a cancer diagnosis and its treatment on financial well-being a of cancer survivor [1, 2]. FT was thought to be prevalent in countries with mainly privately funded healthcare systems, such as the USA [3]. Previous research on financial toxicity and development of FT measures predominately focused in the US healthcare system. However, FT has recently been recognized as a concern even among universal and hybrid healthcare systems, such as in Australia and Canada [4, 5]. Consequently, there is growing research advocating for the development of FT measures specifically designed for these healthcare systems.

Similar to other cancer or treatment-related toxicity, measurement is the first step in evaluating the extent and nature of FT to enable implementation of effective interventions. Instruments designed to measure FT must be grounded on robust frameworks or models and must also demonstrate adequate psychometric properties, such as validity, reliability, and relevant clinical and research utility. Psychometric analysis is a systematic approach to evaluating the quality of measurement instruments [6], providing researchers and practitioners confidence in using validated measures by ensuring that the data collected accurately represents the constructs of interest and supports sound decision-making and research conclusions [7]. However, the diversity in conceptual background, terminology, and contextual factors surrounding FT presents challenges of comparing and quantifying the prevalence of subjective FT. Similarly there are limited cancer-specific measures of FT suitable for universal and hybrid healthcare systems, and those that exist have yet to be available long enough to be psychometrically validated [8]. Standardizing the measurement of FT remains a highly intricate task because the term FT is broad, further complicated by the variations in the experiences of FT due to the different healthcare systems worldwide [9]. To address these challenges, we aim to review existing measures of FT and the conceptual models with the aim of addressing inconsistency in measuring subjective FT.

In recent years, there has been a growing focus among researchers on the multifaceted concept of cancer subjective financial toxicity [2, 10]. This attention has led to efforts to develop frameworks and models aimed at understanding and measuring subjective financial toxicity in a standardized manner. One significant development in this area is the conceptual framework proposed by Altice and colleagues [11]. Their framework introduced a fundamental groundwork for outlining three primary domains essential for understanding subjective financial toxicity, which are material conditions, psychological responses, and coping behaviours [11]. This framework provided a foundational structure for subsequent research in the field. Building upon Altice et al.’s work, Witte and colleagues [9] expanded the framework further by identifying six subdomains that contribute to a comprehensive understanding of subjective financial toxicity in cancer survivors modifiable to a universal healthcare system. These subdomains include active financial spending, utilization of passive financial resources, psychosocial responses, seeking support for financial assistance, coping with the costs of care, and adapting one’s lifestyle to manage financial burdens [9]. Both frameworks underscore the importance of developing instruments for measuring financial toxicity grounded in these domains and subdomains. However, the focus of this systematic review is on the six subdomains proposed by Witte et al. [9] due to their comprehensive coverage of subjective financial toxicity, highlighting perceptions, and responses to financial distress. Witte et al. [9] derived these subdomains from an analysis of 352 different questions in existing literature, providing a detailed overview of the subjective financial toxicity concept and paving a way for the standardization in measurement development.

Consequently, the aims of this systematic review were to (1) identify full measures designed explicitly for assessing FT in cancer survivors and appraise, compare, and summaries their psychometric properties using Consensus-Based Standards for the Selection of Health Measurement Instruments (COSMIN), and (2) provide an analysis of the six domains of FT covered in these measures to better understand the multidimensionality of FT. It was expected that the findings from this systematic review will provide directions for future development of FT instruments.

## Methods

### Protocol and registration

This systematic review was registered with the International Prospective Registry of Systematic Reviews (PROSPERO; Registration ID: CRD42021285726); conducted according to the Cochrane Handbook for Systematic Reviews [12] and COSMIN methodology [13]; and reported according to the Preferred Reporting Items for Systematic Reviews and Meta-analysis (PRISMA) Statement [14].

### Eligibility criteria

Studies were included if they (1) focused on individuals with cancer of any type or stage, (2) focused on cancer-specific measures of FT, (3) were descriptive quantitative studies published in peer-reviewed journals on, or after, the 1 January 2000, (4) described and evaluated the feasibility, validity, and reliability of cancer-specific full measures for assessing FT, and (5) were published in English. Studies were excluded if they were published in another language other than English and were unable to be translated via Google Translate or colleagues, and where full texts were not available online. Studies were further excluded if they described and evaluated sub-scales and screening measures of FT. Mixed methods reviews were included if quantitative data was reported separately.

### Search strategy

MEDLINE, CINAHL, Web of Science, and Cochrane CENTRAL databases were searched from 1 January 2000 to 12 July 2023. The systematic search strategy was based on the following concepts: cancer AND financial toxicity AND cancer survivor AND instrument. A manual search of reference lists of the included studies was conducted until 16 July 2023 to identify articles not found through the database searches.

### Study selection

To eliminate excessive copies of articles, an automated de-duplication of articles was conducted by one author (LT) using Endnote Software (Endnote 20, Clarivate, version 20.2, 2021). Manual text-mining in Endnote was conducted by one author (LT), whereby irrelevant terms were searched in titles and abstracts to identify studies for exclusion, such as *qualitative study*, *systematic review*, *narrative review*, *protocol*, and *conference*. Screening of titles and abstracts, then full texts was completed by two authors independently (LT and MC) using Covidence software (Covidence Systematic Review Software, Veritas Health Innovation, 2021). For any discrepancies in study selection or assessment, authors (LT and MC) discussed their reasons for inclusion or exclusion and worked towards a consensus to include or exclude the study.

### Data extraction, analysis, and quality appraisal

Data extraction was undertaken by one author (LT) and checked for accuracy by a second author (MC), with disagreements managed by discussion among authors. Data extracted in tabular format included study and population characteristics, FT tool characteristics, and psychometric properties. Assessment of study quality was conducted independently by two authors (LT and MC) using the COSMIN Risk of Bias tool [13]. The COSMIN Risk of Bias Checklist compromises standards on design requirements and preferred statistical methods categorized in boxes per measurement properties [13]. Boxes 1 and 2 focus on content validity, and Boxes 3–5 are for structural validity, internal consistency, and cross-cultural validity/measurement invariances [6]. Lastly, Boxes 6–10 address the measurement properties of reliability, measurement error, criterion validity, and hypothesis testing for construct validity and responsiveness [6]. The overall study quality for reliability or measurement error standards was rated as very good, doubtful, inadequate, or not applicable, and the lowest rating of any standard was taken as the final rating [15]. Disagreements were resolved through a systematic and collaborative process between authors (LT and MC) to reconcile differing opinions to reach a consensus.

For each FT tool, the 13 psychometric properties (content validity, relevance, comprehensiveness, comprehensibility, structural validity, internal consistency, cross-cultural validity, measurement invariance, reliability, measurement error, criterion validity, construct validity, and responsiveness) were assessed to determine the quality of the measures [16]. A final rating was determined as sufficient (does meet criteria), insufficient (does not meet criteria), inconsistent (studies report conflicting results as to whether criteria is adequately met), or indeterminate (not enough data to make a decision) [16].

The certainty in the psychometric properties for each FT tool was assessed using the modified Grading of Recommendations Assessment, Development and Evaluation System (GRADE) approach [17]. Two authors (LT and MC) independently generated modified GRADE ratings, and disagreements were resolved via consensus between the authors (LT and MC). The overall GRADE level of certainty in the psychometric property was rated as high, moderate, low, or very low. GRADE ratings were downgraded according to the risk of bias, inconsistency, imprecision, and indirectness using the following methods: risk of bias (downgraded by one, two, or three levels if the serious, very serious, or extremely serious risk of bias, respectively), inconsistency (downgraded by one or two levels if serious or very serious inconsistency, respectively), imprecision (downgraded by one or two levels if between 50 and 100 or <50 study subjects, respectively), and indirectness (downgraded by one or two levels if serious or very serious indirectness, respectively) [6].

### Data synthesis

Study characteristics, risk of bias, FT tool psychometric properties, and GRADE ratings were synthesized narratively in tabular and text format. Domains of FT covered in each of the tools were compared with the six domains of FT recommended by Witte and colleagues [9].

## Results

**Fig. 1:**
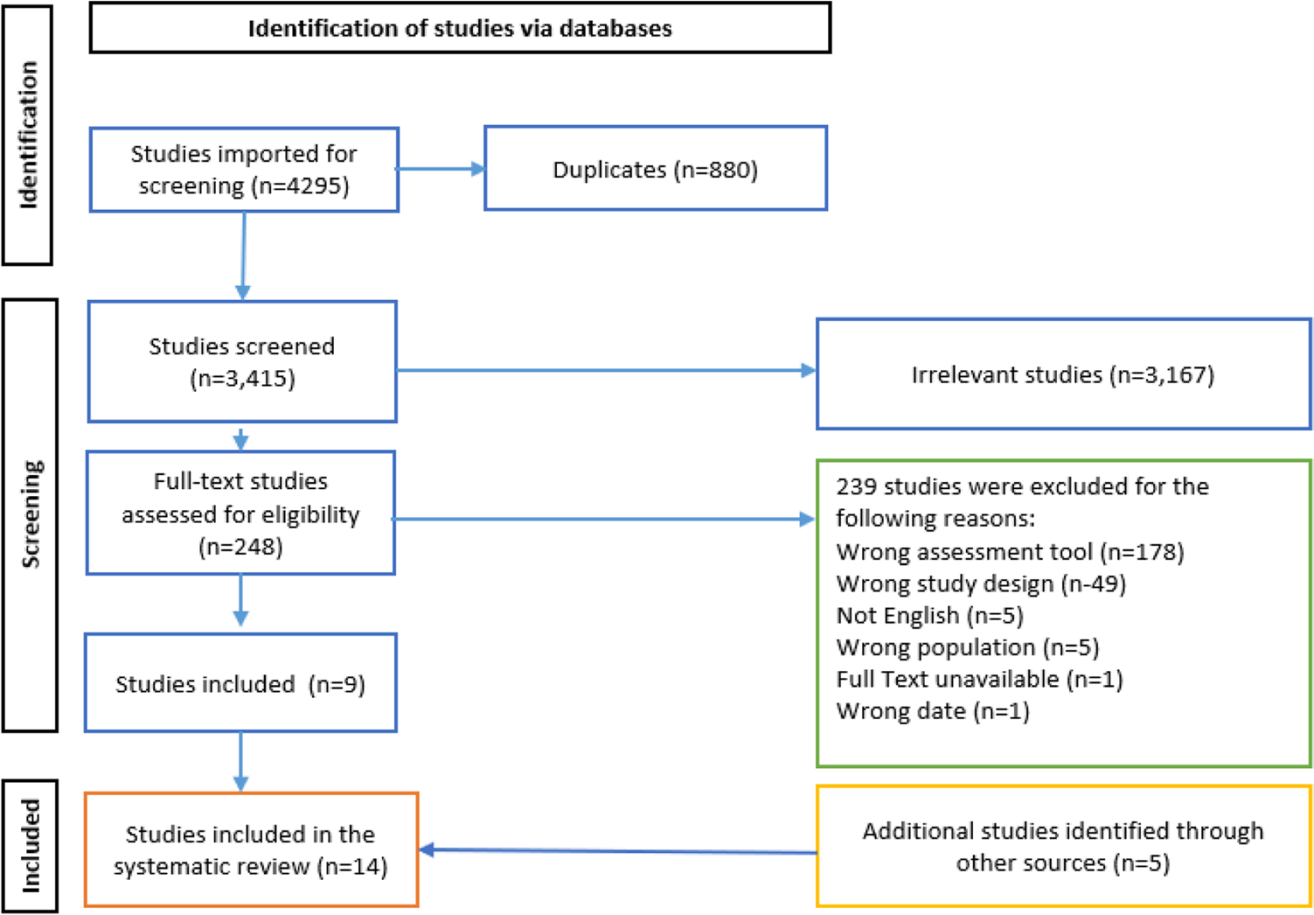
Preferred Reporting Items for Systematic Reviews and Meta-Analyses (PRISMA) flow chart outlining the selection of studies assessing measures of FT in cancer survivors

Of the 6865 records identified, 19 studies were eligible for inclusion, as identified in Fig. 1.

### Study and population characteristics

Article characteristics are summarized in Table 1. Nineteen studies were included and comprised 8582 participants in total, with sample sizes ranging from 12 to 4297 participants per study. The 19 studies represented six measures of cancer FT. Fourteen studies were based on the Comprehensive Score for Financial Toxicity–Functional Assessment of Chronic Illness Therapy (COST-FACIT) Version1 (v1) [4, 20, 21, 23, 25, 27, 28, 35] and Version 2 (v2) [18, 19, 21, 24, 26, 29]. The original COST-FACIT version (V1) consists of 11 items, and the most recent second version (V2) has 12 items. A total score is computed from the sum of items 1 through 11 for either version of the scale (excluding item 12 for V2 of the scale) [36]. The remaining five studies were based on the following measures: the Patient-Reported Outcome for Fighting Financial Toxicity (PROFFIT) measure [32], the Financial Index of Toxicity (FIT) measure [31], the Subjective Financial Distress Questionnaire (SFDQ) [30], the Hardship And Resources with Distress Survey (HARDS)-[34], and the Economic Strain and Resilience in Cancer (ENRICh-Spanish)-[33]. The Enrich-English tool development study was not included in the review because it did not meet inclusion criteria due to only being published as a conference poster. All measures used a five-point Likert scale, and the total number of items ranged from 9 to 16. Three studies were conducted in India [19, 24, 30], the USA [21, 22, 33], and China (includes the mainland and Hong Kong) [18, 29, 34], two studies in Italy [26, 32], and one study each in Australia [4], Brazil [20], Japan [23], Turkey [28], Canada [31], Korea [35], Tunisia [25], and Iran [27]. Most studies focused on multiple cancers except two, which only included people with head and neck cancer [19, 30]

**Table 1:** Study and population characteristics for the included studies assessing cancer-related financial toxicity measures

Citation	PROM	Country/language	Population description	Treatment type and setting	Sample size	No of items/mode of administration	Total score range
The Comprehensive Score for Financial Toxicity–Functional Assessment of Chronic Illness Therapy (COST-FACT) Version 1 and 2							
Chan [18]	COST-FACIT V-2	Country: Hong Kong Language: Traditional Chinese and English	Age: mean 60 years (SD 11) Female: 64% Cancer type: Mixed solid tumours (94%), mixed hematological (4%), other (2%)	Setting: Hospital outpatient clinic Treatment: Surgery (65%), RT (45%), CT (82%), targeted therapy (41%), HRT (22%), immunotherapy (3%)	640	No. of items: 12 Mode of administration: Self-administered questionnaires	0–44 Five-point Likert scale
Dar [19]	COST-FACIT V-2	Country: India Language: Hindi and English	Age: mean 50 years (SD 17) Female: 17% Cancer type: head and neck (100%)	Setting: Radiation oncology outpatient department Treatment: RT (17%), RT + surgery (48%), RT + CT (21%), RT + CT + surgery (14%)	29	No. items: 11 Mode of administration: Recorded face-to-face interview	0–44 Five-point Likert scale
de Alcantara [20]	COST-FACIT V-1	Country: Brazil Language: Brazilian Portuguese and English	Age: mean 56 years Female: 60% Cancer type: not reported.	Setting: Hospital outpatient department Treatment: CT (100%)	126	No. of items: 11 Mode of administration: Self-administered questionnaires	0–44 Five-point Likert scale
de Souza [21]	COST-FACIT V-1	Country: USA Language: English	Age: median 60 years (range 24–84) Female: 45% Cancer type: Mixed solid tumours 86%, mixed hematological (3%), other (11%)	Setting: Oncology outpatient Treatment: Not reported	155	No. of items: 11 Mode of administration: Semi-structured qualitative interviews	0–44 Five-point Likert scale
de Souza [22]	COST-FACIT V-2	Country: USA Language: English	Age: mean 58 years (SD11) Female: 58% Cancer type: Mixed solid tumours 89%, other (11%)	Setting: Cancer centres Treatment: CT (oral, intravenous, or both; 100%)	233	No. of items 11 Mode of administration: Interviews	0–44 Five-point Likert scale
Durber [4]	COST-FACIT V-1	Country: Australia Language: English	Age: median 63 years (range 19–88) Females: 54% Cancer type: Mixed solid tumours (100%)	Setting: Oncology outpatient Treatment: current evidence of disease with or without current treatment	257	No. of items 11 Mode of administration: self-administered questionnaires	0-44 Five-point Likert scale
Honda [23]	COST-FACIT V-1	Country: Japan Language: English and Japanese	Age: median 65 years (range 30–72) Females: 73% Cancer type: Mixed solid tumours (100%)	Setting: Oncology outpatient Treatment: CT + targeted therapy (73%), not receiving treatment (27%)	12	No. of items 11 Mode of administration: Self-administered questionnaires	0–44 Five-point Likert scale
Joshi [24]	COST-FACIT V-2	Country: India Language: English, Hindi, and Marathi	Age: not reported Females: not reported Cancer types: Mixed solid tumours (100%)	Setting: Uro-oncology department Treatment: not reported	20	No. of items 11 Mode of administration: Self-administered questionnaires and interview	0–44 Five-point Likert scale
Mejri [25]	COST-FACIT V-1	Country: Tunisia Language: Arabic	Age: median 52 years (SD 12) Females: 71% Cancer type: Mixed solid tumours (90%), other (10%)	Setting: Oncology clinic Treatment: CT, surgery, RT	179	No. of items: 11 Mode of administration: Questionnaire and cognitive interview	0–44 Five-point Likert scale
Ripamonti [26]	COST-FACIT V-2	Country: Italy Language: Italian	Age: mean 61 years (SD 13) Females: 47% Cancer types: Mixed solid tumours (73%), mixed hematological (23%), other (2%)	Setting: Oncology outpatient Treatment type: Surgery (2%), RT (25%), CT (63%), HRT (24%), targeted therapy (22%), immunotherapy (20%)	118	No. of items 11 Mode of administration: Self-administered questionnaires	0–44 Five-point Likert scale
Sharif [27]	COST-FACIT V-1	Country: Iran Language: Persian	Age: mean 50 years (SD 14) Females: 46% Cancer: Not reported	Setting: Oncology clinics and research centres Treatment: 77% under treatment, (not specified), 12% completed treatment, and 10% newly diagnosed	407	No. of items: 11 Mode of administration: Questionnaire	0–44 Five-point Likert scale
Shim [27]shi	COST-FACIT V-1	Country: Korea Language: Korean and English	Age: mean 50 years (SD 9) Females: 100% Cancer type: Breast (100%)	Setting: Outpatient breast cancer/oncology clinic Treatment type: Disease-free survivors. Treatment completed within the past 5 years. CT (45%), RT (75%), and HRT (79%)	4297	No. of items 11 Mode of administration: Interviews	0–44 Five-point Likert scale
Urek [28]	COST-FACIT V-1	Country: Turkey Language: Turkish and English	Age: mean 56 years (SD 12) Females: 49% Cancer types: Mixed solid tumours (76%), mixed hematological (24%)	Setting: Inpatient Treatment: CT (100%)	316	No. of items 11 Mode of administration: Face-to-face interviews	0–44 Five-point Likert scale
Yu [29]	COST-FACIT V-1	Country: China Language: Chinese and English	Age: mean 57 years (SD 9) Females: 54% Cancer types: Mixed solid tumours (90%), other (10%)	Setting: Inpatient Treatment: CT (59%), surgery (23%), CT and surgery (18%)	440	No. of items 11 Mode of administration: questionnaires by a trained nurse	0–44 Five-point Likert scale
Subjective Financial Distress Questionnaire (SFDQ)							
Dar [30]	SFDQ	Country: India Language: English	Age: not reported Female: 14% Cancer type: Head and neck (100%)	Setting: Radiation oncology outpatient department Treatment type: RT (12%), RT + surgery (59%), RT + CT (31%), RT + CT + surgery (16%)	142	No. of items: 14 Mode of administration: Face-to-face interviews	0–28 Five-point Likert scale
Financial Index of Toxicity (FIT)							
Hueniken [31]	FIT	Country: Canada Language: English	Age: median 62 years (range 26–89) Females: 23% Cancer type: Head and neck (100%)	Setting: Oncology outpatient Treatment: CT + RT (40%), RT (31%), surgery (14%), surgery + RT and/or CT + RT (14%), other/unknown (1%)	430	No. of items 9 Mode of administration: Self-administered questionnaires	0–100 Five-point Likert scale
The Patient Reported Outcome for Fighting Financial Toxicity of cancer (PROFFIT)							
Riva [32]	PROFFIT	Country: Italy Language: English and Italian	Age: median 59 years (range 29–83) Females: 59% Cancer types: Mixed solid tumours (100%)	Setting: Clinical oncological centres Treatment: CT (68%), target agents (11%), immunotherapy (15%), HRT (3%), RT (1%)	184	No. of items: 16 Mode of administration: administered either as paper document or as a tablet digital version	0–100 Five-point Likert scale
Economic Strain and Resilience in Cancer (ENRICh)							
Shi [33]	ENRICh-Spanish	Country: USA Language: UN-Spanish	Age: mean 50 years (SD 14) Females: 65% Cancer type: Mixed solid tumours (83%), mixed hematological (9%), other (8%)	Setting: Ambulatory oncology care Treatment: CT (100%)	77	No. of items: 15 Mode of administration: Survey and cognitive qualitative interviews	0–10 Five-point Likert scale
Hardship And Recovery with Distress Survey (HARDS)							
Liu [34]	HARDS	Country: China Language: Not reported	Age: not reported Females: 40% Cancer type: Mixed solid tumours (83%), other (17%)	Setting: Not reported Treatment: Not reported	518	No. of items: 10 Mode of administration: Not specified	10–50 Five-point Likert scale

*CT* chemotherapy, *GI* gastrointestinal, *No.* number, *RT* radiotherapy, *SD* standard deviation, *HRT* Hormonal Replacement Therapy

### Domains of subjective financial toxicity

The six measures of FT that reviewed the six domains of FT as outlined by Witte and colleagues [11] are presented in Table 2. Coping and support seeking under behavioural responses was the domain with the best coverage (included in the SFDQ, HARDS, ENRICh-Spanish, and PROFFIT). The SFDQ, HARDS, and ENRICh-Spanish included all six domains of subjective FT (active financial spending, use of passive financial resources, psychosocial responses, support seeking, coping with care, and coping with one’s lifestyle). The COST-FACIT measure included material and psycho-social domains. The FIT measure included one component of the behavioural domain (financial resources) and the psycho-social domain. Lastly, the PROFFIT measure included material spending and one component of the behavioural domain (support seeking).

**Table 2:** Number of items in each cancer-related financial toxicity measure that cover each of the six domains of financial toxicity

Measures	Total items	Number of items per domain					
		Material		Psycho-social	Behavioural		
		Financial spending	Financial resources	Affect	Support seeking	Coping care	Coping lifestyle
COST v1	11	1	2	8	0	0	0
COST v2	11	1	2	8	0	0	0
SFDQ	14	5	2	3	2	1	1
FIT	9	0	2	7	0	0	0
PROFFIT	16	9	4	0	3	0	0
ENRICh-Spanish	15	4	2	1	4	3	1
HARDS	10	2	2	2	1	2	1

Six domains of financial toxicity Witte and Colleagues [11]. Comprehensive Score for Financial Toxicity–Functional Assessment of Chronic Illness Therapy (COST-FACIT), Patient-Reported Outcome for Fighting Financial Toxicity (PROFFIT), Financial Index of Toxicity (FIT), (4) Subjective Financial Distress Questionnaire (SFDQ), Hardship And Resources with Distress), Economic Strain and Resilience in Cancer (ENRICh-Spanish)

### Methodological quality of included studies

The COSMIN quality of study development and validation measures of financial toxicity are summarized in Table 3. Almost all studies were rated as having very good internal consistency (*n* = 18 studies, 95% [4, 18-29, 31-34, 36]), =hypothesis testing (*n* = 17, 89% [4, 18-29, 31, 32, 34, 36]), and criterion validity (*n* = 17 studies, 89%)[4, 18-20, 22-29, 31-34, 36]). Ratings were mostly inadequate for the quality of the development of FT measures (*n* = 13 studies, 68% [4, 18, 19, 21-23, 25-28, 32, 33, 36]), measurement error (*n* = 13, 68% [18-28, 35, 36]), content validity (*n* = 12, 63% [4, 18-21, 25-29, 31, 32]), cross-cultural validity (*n* = 11, 58% [18, 20, 23, 26-29, 31, 32, 35, 36]), and structural validity (*n* = [4, 18, 19, 22-24, 26, 27, 35]). Ratings for reliability were inadequate or doubtful (*n* = 11, 58% [19-23, 25-27, 33, 35], and adequate: *n* = 8, 42% [4, 18, 24, 28, 29, 31, 32, 34, 36]).

**Table 3:**
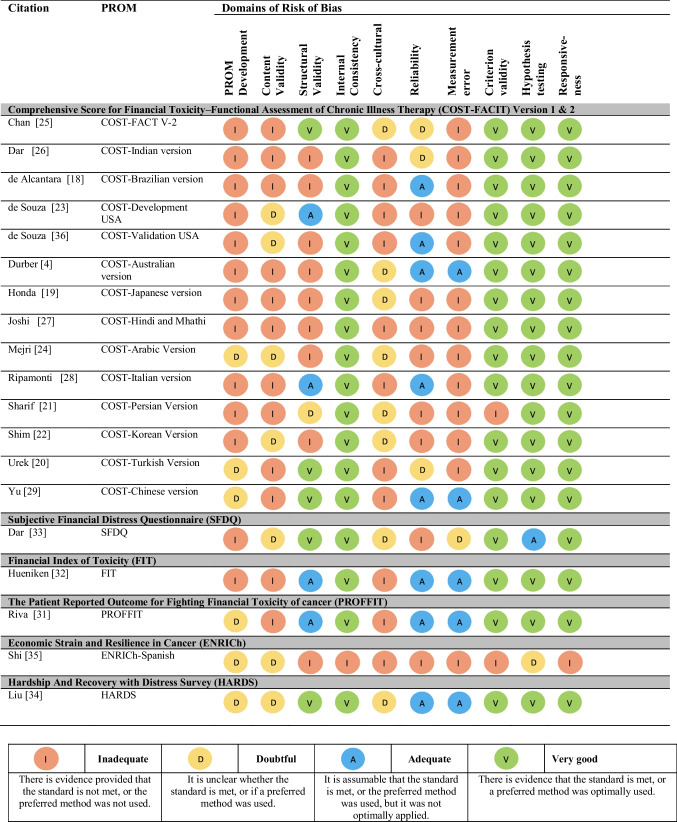
COSMIN risk of bias assessment for the included studies

### Psychometric properties

The psychometric properties of the included studies are presented in Table S2. Internal consistency was presented as Cronbach’s alpha for all studies [4, 18–35]. The 11 studies that assessed structural validity used either exploratory factor analysis (EFA) [18, 21, 26, 31, 32] or confirmatory factor analysis (CFA) [20, 27–30, 34]. Six studies assessed reliability, all of which used intraclass correlations [4, 18, 22, 29, 34, 35]. Six studies assessed hypothesis testing using Pearson’s correlation coefficient (*r*) [4, 18, 22, 26, 29, 35] and two studies assessed cross-cultural validity using probability (*p* value) [20, 27]. No studies assessed measurement error and criterion validity.

### Overall rating and quality of evidence for each measure

There was moderate quality evidence for sufficient relevance for three measures (SFDQ, PROFFIT, HARDS; Table 4). The overall content validity GRADE rating varied considerably with (COST-FACIT, SFDQ, PROFFIT, HARDS, ENRICH-Spanish ranging from very low to insufficient while FIT; GRADE was rated Low. Regarding internal structure, HARDS had sufficient moderate quality evidence for all measurement properties. The SFDQ had sufficient ratings for two of the four internal structure measurement properties (structural validity and internal consistency), with the quality of the evidence rating very low. For measurement properties, all measures except for ENRICH-Spanish had sufficient hypothesis testing for construct validity and responsiveness, while the quality evidence for COST-FACIT rated high. FIT and PROFFIT measures had sufficient moderate quality evidence for reliability. The HARDS tool had sufficient moderate quality evidence for criterion validity. All tools had indeterminate measurement error.

**Table 4:** Psychometric property overall ratings and GRADE ratings for cancer-related financial toxicity measures

	COST-FACIT		SFDQ		FIT		PROFFIT		HARDS		ENRICh-Spanish	
	Overall rating	GRADE rating	Overall rating	GRADE rating	Overall rating	GRADE rating	Overall rating	GRADE rating	Overall rating	GRADE rating	Overall rating	GRADE rating
Content validity												
Overall content validity	±	Low	±	Very low	-	Low	±	Very Low	±	Very Low	±	Very Low
Relevance	±	Moderate	+	Moderate	±	Low	+	Moderate	+	Moderate	?	Very low
Comprehensiveness	±	Low	±	Very low	-	Low	±	Very low	±	Very low	±	Very low
Comprehensibility	±	Moderate	±	Low	±	Low	-	Moderate	±	Low	±	Very low
Internal structure												
Structural validity	?	Moderate	+	Very low	?	Moderate	?	Low	+	Moderate	?	Low
Internal consistency	?	Moderate	+	Very low	?	Moderate	?	Moderate	+	Moderate	?	Low
Cross-cultural validity	?	Moderate	?	Very low	?	Low	?	Low	+	Moderate	?	Low
Measurement invariance	?	Moderate	?	Very low	?	Moderate	?	Low	+	Moderate	?	Low
Other measurement properties												
Reliability	?	Low	?	Very low	+	Moderate	+	Moderate	?	Moderate	?	Low
Measurement error	?	Moderate	?	Very low	?	Low	?	Low	?	Moderate	?	Low
Criterion validity	?	Moderate	?	Very low	-	Moderate	?	Moderate	+	Moderate	?	Low
Hypothesis testing for construct validity	+	High	+	Very low	+	Moderate	+	Moderate	+	Moderate	?	Low
Responsiveness	+	High	+	Very low	+	Moderate	+	Moderate	+	Moderate	?	Low

Sufficient (+), insufficient (–), inconsistent (±), or indeterminate (?)

GRADE: Grading of Recommendations Assessment, Development and Evaluation system (High: high certainty/confidence in the measurement property rating; Moderate: moderate certainty/confidence in the measurement property rating; Low: low certainty/confidence in the measurement property rating; Very low: very low certainty/confidence in the measurement property rating)

*Estimate of the measurement property refers to the pooled or summarized result of the measurement property of a PROM

## Discussion

This systematic review highlighted an ongoing need for a validated and comprehensive measure of subjective financial toxicity. . The findings of this review complement those of a recently published systematic review [8], which also conducted a psychometric property analysis of cancer-specific FT instruments and subscales using the COSMIN framework. In contrast, this current review primarily focused on identifying cancer-specific full instruments explicitly designed to measure FT and assessing the psychometric properties using the COSMIN methodology. Additionally, this current review aimed to assess the comprehensiveness of the subjective FT measures by comparison with the six domains of FT as outlined by Witte and Colleagues [9].

This review found the HARDS measure was the most comprehensive tool for assessing FT, demonstrating satisfactory ratings for psychometric analysis, and effectively covering all domains of FT. However, the HARDS measure is newly developed in China; thus, its validation in various cultural and healthcare settings has not been established. On the other hand, COST-FACIT emerged as the most thoroughly evaluated measure, being the sole measure to provide high-quality evidence suitable for hypothesis testing in terms of construct validity and responsiveness. But it is notably limited in several critical areas, including the evaluation of internal structure (including structural and cross-cultural validity), internal consistency, reliability, measurement error, and criterion validity.

Cross-cultural validity is of particular importance as it focuses on evaluating the relevance and meaningfulness of the instrument across diverse cultural contexts, taking into account potential cultural biases, language differences, and variations in cultural norms, values, and experiences [6]. The COSMIN framework recommends specific statistical methods for establishing cross-cultural measurement invariance, including Confirmatory Factor Analysis (CFA) and Differential Item Functioning (DIF) [6]. Notably, three COST-FACIT studies [19, 21, 26] used Exploratory Factor Analysis (EFA) to establish measurement invariance, which is not the most appropriate method for this purpose. EFA lacks the capability to compare the goodness-of-fit of factor models across different groups, as achieved through indices like the Comparative Fit Index (CFI), which is crucial for assessing measurement invariance [37].

Additionally, five studies [4, 18, 22–24] of the COST-FACIT measure did not report evidence on the measurement invariance, and one study [20] reported negative CFA. The absence of such evidence or negative CFA findings regarding measurement invariance may imply that the relationship between items and latent constructs varies across different cultures [37]. Yoon and Colleagues [38] argue that several factors can contribute to a negative CFA in terms of cross-cultural validity, such as variations in the conceptualization of the construct across cultures or even cultural differences in the underlying meaning of the items themselves. It is important to emphasize that using the same instruments with culturally diverse groups necessitates testing measurement invariance across these groups, even if they share a common language. For instance, despite both Australia and the USA being English-speaking countries, the experiences of FT differ due to disparities in healthcare systems [4]. Thus, different countries, despite a shared language, may exhibit structural and cultural distinctions that result in varying underlying factor structures for the same instrument [37].

Given the widespread use of the COST-FACIT measure, future studies should be conducted to ascertain whether the cultural validity of measures of FT is measured the same way across different cultures and healthcare systems to ensure measures of FT accurately capture FT across diverse cultural groups. Hence, Kulhawy-Wibe and Colleagues [39] and Regnault and Herdman [40] argue that it is essential that clear and rigorous methods are adapted in the translation and cultural adaptation of patient-reported measures to ensure that the latent trait of the measure (in this case, FT) is being measured in the same way across cultures. Although the COST-FACIT showed an adequate development process, it is limited in its comprehensive assessment of the six key domains as proposed by Witte and colleagues [9]. Specifically, it lacks coverage of crucial aspects such as financial coping and support-seeking domains, which are integral for capturing cancer survivors’ experiences of subjective FT. From the literature cancer survivors unable to bear the cost of cancer treatment may resort to various coping mechanisms, including non-compliance with treatment, reduced spending on essentials like food, and borrowing money [30]. It is worth noting that the development of the COST measure predates the recommendation to incorporate the six domains of FT, contributing to its limitations in encompassing these critical dimensions.

Another measure that provided adequate data on psychometric properties was the FIT measure, developed in Canada by Hueniken and Colleagues [31], to measure FT in head and neck cancer survivors. Developed and validated in a universally funded healthcare system, the FIT measure is brief. The FIT measure may not be appropriate for countries with cancer survivors receiving care in private health systems. Future studies are required to assess the applicability and validity of FIT to other cancer streams and its applicability in assessing FT in a privately funded or mixed-funded healthcare system. Other measures identified in the review are the ENRICH Spanish, which was developed in the US among Spanish speaking cancer survivors and the SFDQ and PROFFIT which were not developed in an English-speaking developed country. The measures were developed in India and Italy where these countries’ socioeconomic contexts and healthcare systems may differ significantly from other healthcare systems. This stipulation is supported by Zhu and Colleagues [8], who emphasized the strong connection between financial toxicity (FT) and broader social determinants of economic circumstances, including healthcare policies, healthcare systems, insurance structures, and the level of economic development. These factors can not only influence cancer survivors’ perceived FT levels but also play a role in shaping the sources of FT. Nevertheless, out of the six measures reviewed, the SFDQ, HARDS, and ENRICH Spanish are the only measures that comprehensively cover all six domains of subjective FT.

Finally, the SFDQ is specifically designed to measure FT in patients undergoing radiation therapy. There are several limitations to consider when using the SFDQ to measure FT in patients undergoing different cancer treatment modalities, such as the greater emphasis on out-of-pocket expenses specific to radiation treatment, which may not adequately capture the FT associated with other therapies [30, 41]. Furthermore, the SFDQ faces constraints due to a lack of validation in diverse treatment populations, and its reliability and validity in patients receiving different cancer treatments have not been extensively investigated, thereby limiting its accuracy and meaningful interpretation [8].

By applying the COSMIN guidelines, the PROFFIT and the HARDS measures demonstrated high-quality evidence for content validity compared to other measures. COSMIN criteria requires evaluation of relevance, comprehensiveness, and comprehensibility in the assessment of content validity [15]]. However, it is important to note a gap in the evidence regarding content validity since cognitive interview testing was not conducted on the final versions of the COST-FACIT, FIT, and SFDQ measures to assess their relevance and comprehensiveness. One of the COSMIN requirements for a sufficient rating in content validity mandates that studies also consult with the target population, in this case, cancer survivors, to assess relevance, comprehensiveness, and comprehensibility [15]. Additionally, there was a risk of bias across various domains, and the quality of evidence in most of the included studies was generally rated as very low to low, which poses challenges to the validity, reliability, and overall credibility of the findings [16].

Overall, the evidence for the quality of the measures and inclusion of domains of subjective financial toxicity differed considerably. No measure reached an excellent level for overall quality but was mostly rated as sufficient. However, the systematic review highlighted the strides made in tailoring measures of FT to suit specific countries, reflecting progress in addressing localized experiences of subjective FT. Despite significant progress in this area, challenges remain, particularly concerning the variability in measures of subjective financial distress across studies. This variability highlights the need for a consistent definition of subjective FT and standardized measurements capable of thoroughly assessing the multifaceted aspects of subjective FT. Understanding these systemic factors is essential for developing targeted interventions to alleviate financial burdens and improve the overall well-being of individuals facing cancer diagnosis and treatment. Rigorous validation and testing are essential to ensure the applicability of these measures across diverse cancer types, healthcare systems, and cultural contexts, while also considering factors such as translation and local nuances. Future research should aim to enhance the cultural sensitivity of these measures to promote inclusivity and accuracy in assessing FT experiences among individuals from cultural backgrounds. Another pressing priority is the creation of practical screening tools demonstrating high predictive accuracy through correlation with scores for all domains of FT in clinical practice. Integrating the screening for cancer survivors at risk of FT as a standard practice is essential, given the multitude of consequences associated with FT [41]. It is worth noting that the FT measures identified in this review may not be the most suitable for use as screening tools due to their length and the time required for their administration. For instance, Prasad and colleagues [42] argue that measures like COST-FACIT can be cumbersome and challenging to use for screening cancer survivors at risk of developing FT. Nevertheless, the argument presented by Beauchemin and colleagues [43] highlights the critical importance of early identification of FT in mitigating catastrophic financial losses and addressing existing disparities in healthcare delivery. This highlights the imperative for future tool development.

### Strengths and limitations

This systematic review used robust methodology and stringent adherence to the PRISMA statement [14] to provide a current and comprehensive analysis of available literature. This review has succeeded in discussing the many limitations of the included studies regarding the development and assessment of FT tools to best guide future research and practice. In addition, numerous limitations regarding the conduct of this review were present. It is possible that the exclusion of grey literature and other databases may have excluded relevant studies; however, this is unlikely due to the rigorous search strategy used and manual searching of references lists of relevant literature. Only English studies were included, which might have led to under-representation of non-English countries. The review relied on COSMIN to analyse the FT tools, which is complex, required expertise in psychometrics, and is subjective.

## Conclusion

This review emphasizes the need for validated multidimensional FT measures that can be applied across various cancer types, healthcare settings, and cultural backgrounds. Furthermore, a need to develop practical screening tools with high predictive ability for FT is highly important, considering the significant consequences of FT.

### Supplementary Information

Below is the link to the electronic supplementary material.

Supplementary file1 (DOCX 14.4 KB)

Supplementary file2 (DOCX 33.7 KB)

## Data Availability

Not applicable
