# Supplementary material for: Measures of financial toxicity in cancer survivors: a systematic review
Source: Support Care Cancer. 2024 Jun 4;32(7):403. doi: 10.1007/s00520-024-08601-4 (PMC11147933; doi:10.1007/s00520-024-08601-4)
Supplement: Supplementary file 1 — Supplementary file1 (DOCX 14.4 KB) [file 520_2024_8601_MOESM1_ESM.docx]

**Supplementary Table S1. Systematic search terms used to locate studies examining measures of cancer-related financial toxicity.**

| Database | Search Strategy |
| --- | --- |
| **Medline** | TI cancer* OR TI Neoplasm* OR TI Tumor* OR TI Tumour* OR TI Oncolog* OR TI Carcinoma* OR TI Hematolog* OR TI Haematolog* AB Cancer* OR AB Neoplasm* OR AB Tumor* OR AB Tumour* OR AB Oncolog* OR AB Carcinoma* OR AB Hematolog* OR AB Haematolog* MH “neoplasms” TI Financial* OR TI “Economic burden*” OR TI “Economic hardship*” OR TI Expense* OR TI “Productivity loss*” OR TI “out-of-pocket” OR TI “co-payment*” OR TI Income* OR TI Poverty* AB Financial* OR AB “Economic burden*” OR AB “Economic hardship*” OR AB Expense* OR AB “Productivity loss*” OR AB “out-of-pocket” OR AB “co-payment*” OR AB Income* OR AB Poverty* MH "financial stress" TI Instrument* OR TI Scale* OR TI Questionnaire* OR TI Survey* OR TI Tool* OR TI Assessment* AB Instrument* OR AB Scale* OR AB Questionnaire* OR AB Survey* OR AB Tool* OR AB Assessment* MH "Surveys and Questionnaires" TI “Cancer Survivor*” OR TI “Cancer Patient*” AB “Cancer Survivor*” OR AB “Cancer Patient*” MH “cancer survivors” |
| **Cochrane** | (Cancer*):ti,ab,kw OR (Neoplasm*):ti,ab,kw OR (Tumor*):ti,ab,kw OR (Tumour*):ti,ab,kw OR (Oncolog*):ti,ab,kw (Carcinoma*):ti,ab,kw OR (Hematolog*):ti,ab,kw OR (Haematolog*):ti,ab,kw MeSH descriptor: [Neoplasms] explode all trees (Financial*):ti,ab,kw OR (“Economic burden*”):ti,ab,kw OR (“Economic hardship*”):ti,ab,kw OR (Expense*):ti,ab,kw OR (“Productivity loss*”) (“out-of-pocket”):ti,ab,kw OR (“co-payment*”):ti,ab,kw OR (Income*):ti,ab,kw OR (Poverty):ti,ab,kw MeSH descriptor: [Financial Stress] explode all trees (Instrument*):ti,ab,kw OR (Scale*):ti,ab,kw OR (Questionnaire*):ti,ab,kw OR (Survey*):ti,ab,kw OR (Tool*):ti,ab,kw (Assessment*):ti,ab,kw MeSH descriptor: [Surveys and Questionnaires] explode all trees (“Cancer Survivor*”):ti,ab,kw OR (“Cancer Patient*”):ti,ab,kw MeSH descriptor: [Cancer Survivors] explode all trees |
| **CINAHL** | TI cancer* OR TI Neoplasm* OR TI Tumor* OR TI Tumour* OR TI Oncolog* OR TI Carcinoma* OR TI Hematolog* OR TI Haematolog* AB Cancer* OR AB Neoplasm* OR AB Tumor* OR AB Tumour* OR AB Oncolog* OR AB Carcinoma* OR AB Hematolog* OR AB Haematolog* MH “neoplasms” TI Financial* OR TI “Economic burden*” OR TI “Economic hardship*” OR TI Expense* OR TI “Productivity loss*” OR TI “out-of-pocket” OR TI “co-payment*” OR TI Income* OR TI Poverty* AB Financial* OR AB “Economic burden*” OR AB “Economic hardship*” OR AB Expense* OR AB “Productivity loss*” OR AB “out-of-pocket” OR AB “co-payment*” OR AB Income* OR AB Poverty* MH "financial stress" TI Instrument* OR TI Scale* OR TI Questionnaire* OR TI Survey* OR TI Tool* OR TI Assessment* AB Instrument* OR AB Scale* OR AB Questionnaire* OR AB Survey* OR AB Tool* OR AB Assessment* MH "Surveys and Questionnaires" TI “Cancer Survivor*” OR TI “Cancer Patient*” AB “Cancer Survivor*” OR AB “Cancer Patient*” MH “cancer survivors” |
| **Web of Science** | “Cancer Survivor*” (Topic) or "Cancer Patient*” (Topic) Instrument* (Topic) or Scale* (Topic) or Questionnaire* (Topic) or Tool* (Topic) or Assessment* (Topic) or Survey* (Topic) TS=(Financial* ) OR TS=(“Economic burden*” ) OR TS=(“Economic hardship*” ) OR TS=(Expense* ) OR TS=(“Productivity loss*” ) OR TS=(“out-of-pocket” ) OR TS=(“co-payment*” ) OR TS=(Income*) OR TS=(Poverty*) Cancer* (Topic) or Neoplasm* (Topic) or Tumor* (Topic) or Tumour* (Topic) or Oncolog* (Topic) or Carcinoma* (Topic) or Hematolog* (Topic) or Haematolog* (Topic) |
