# Supplementary material for: Measures of financial toxicity in cancer survivors: a systematic review
Source: Support Care Cancer. 2024 Jun 4;32(7):403. doi: 10.1007/s00520-024-08601-4 (PMC11147933; doi:10.1007/s00520-024-08601-4)
Supplement: Supplementary file 2 — Supplementary file2 (DOCX 33.7 KB) [file 520_2024_8601_MOESM2_ESM.docx]

**Supplementary Table S2. Psychometric properties of cancer-related financial toxicity measures for each of the included studies.**

| **Author (year)** | **PROM** | **Structural validity** | | **Internal Consistency** | **Reliability** | **Measurement Error** | **Hypothesis testing** | **Cross-cultural validity** | **Criterion validity** |
| --- | --- | --- | --- | --- | --- | --- | --- | --- | --- |
| Chan [25] | COST-Chinese version | NR | | + Cronbach's alpha: 0.86 | Intraclass correlation coefficient: 0.71+ | NR | (r= -0.46-0.47) | NR | NR |
| Dar [26] | COST-Indian version | -  EFA: Chi-square 60.82 | | + Cronbach's alpha: 0.92 | NR | NR | NR | NR | NR |
| de Alcantara[18] | COST-Brazilian version | Confirmatory factor analysis:  +(CFA)-value of—χ2/gl was less than 2: 179.78, p=0.000 | | + Cronbach's alpha: 0.815 | NR | NR | NR | p=<0.001 | NR |
| de Souza [23] | COST-Development USA | -  EFA (Chi-square 7.26) | | + Cronbach's alpha: 0.9 | The intraclass correlation coefficient was 0.47 - | NR | NR | NR | NR |
| de Souza [36] | COST-Validation USA | NR | | + Cronbach's alpha: 0.92 | Intraclass correlation coefficient:0.80 + | NR | r=0.26-0.42 | NR | NR |
| Durber [4] | COST-Australian version | NR | | + Cronbach's alpha: 0.884 | Intraclass correlation coefficient: 0.80 + | NR | r=0.17-0.53 | NR | NR |
| Honda [19] | COST-Japanese version | NR | | + Cronbach's alpha: 0.87 | NR | NR | NR | NR | NR |
| Ripamonti [28] | COST-Italian version | -  EFA  KMO: 0.82, 95% CI: 0.80-0.86 (adequate sample)  - Bartlett's x2: 486.2, p<0.001 | | + Cronbach's alpha: 0.83 | NR | NR | r=0.79 | NR | NR |
| Joshi [27] | COST-Hindi and Mhathi | NR | | + Cronbach's alpha: 0.85 | NR | NR | NR | NR | NR |
| Urek [20] | COST-Turkish Version | +  CFA  RMSEA: 0.054  CFI: 0.925  NFI: 0.857  TLI: 0.914  GFI: 0.888 | | + Cronbach's alpha: 0.92 | NR | NR | NR | NR | NR |
| Yu [29] | COST-Chinese version | +  CFA (CFI =0.86, SRMR=0.08) | | + Cronbach's alpha: 0.85 | Intraclass correlation coefficient: 0.85 + | NR | r=0.20-0.44 | NR | NR |
| Shim [22] | COST-Korean Version | NR | | + Cronbach's alpha: 0.81 | Intraclass correlation coefficient: 0.78 + | NR | r=-0.60 | NR | NR |
| Sharif [21] | COST-Persian Version | + CFA  RMSEA:0.071  SRMR:0.051  CFI:0.966  TLI:0.953  NFI:0.936  IFI:0.967 | | + Cronbach's alpha: >0.70 | NR | NR | NR | p=<0.001 | NR |
| Mejri [24] | COST-Arabic Version | NR | | + Cronbach's alpha: 0.77 | NR | NR | NR | NR | NR |
| Riva [31] | PROFFIT | - EFA (no information) | | + Cronbach's alpha: 0.87 | Intraclass correlation coefficient 0.52 to 0.79 | NR | NR | NR | NR |
| Hueniken [32] | FIT | -  EFA (no information) | | + Cronbach's alpha: 0.77 | Interclass correlation coefficient: 0.70 - | NR | NR | NR | NR |
| Dar [33] | SFDQ | +  CFA  RMSEA:0.045  SRMR:<0.014  CFI:0.92  TLI:0.97 | | + Cronbach's alpha: 0.87 | NR | NR | NR | NR | NR |
| Liu [34] | HARDS | +  CFA  RMSEA:  ≤0.080  GFI: ≥0.900  CFI: ≥0.960  TLI: ≥0.900 | + Cronbach's alpha:  0.83 | | Intraclass correlation, 0.90+ | NR | NR | NR | NR |
| Shi [35] | ENRICh-Spanish | NR | | + Cronbach's alpha: 0.90 | NR | NR | NR | NR | NR |

AUC area under the curve, CFA confirmatory factor analysis, CFI comparative fit index, CTT classical test theory, DIF differential item functioning, ICC intraclass correlation coefficient, IRT item response theory, LoA limits of agreement, MIC minimal important change, RMSEA root mean square error of approximation, SEM standard error of measurement, SDC smallest detectable change, SRMR standardized root mean residuals, TLI Tucker–Lewis index. ± inconsistent results; − unsatisfactory results; + satisfactory results; NR: not reported
